# RIG-I drives protective type I interferon production by glial cells in response to *Neisseria meningitidis* and *Streptococcus pneumoniae* challenge

**DOI:** 10.3389/fimmu.2025.1692421

**Published:** 2025-11-18

**Authors:** Krishna J. Majithia, M. Brittany Johnson

**Affiliations:** Department of Biological Sciences, University of North Carolina at Charlotte, Charlotte, NC, United States

**Keywords:** meningitis, microglia, astrocytes, RIG-I, type I interferons, *Neisseria meningitidis*, *Streptococcus pneumoniae*

## Abstract

**Introduction:**

Bacterial meningitis is a rapidly progressing and often fatal infection of the central nervous system (CNS), characterized by glial cell activation and potent neuroinflammatory responses. While Toll-like receptors have been well-characterized in CNS immunity, the contribution of cytosolic nucleic acid sensors such as retinoic acid-inducible gene I (RIG-I) remains largely undefined. Although RIG-I is classically associated with initiating antiviral responses, including type I interferon (IFN) production, emerging evidence supports its role in sensing bacterial nucleic acids. Building upon prior findings that bacterial RNA can activate RIG-I in glial cells, we sought to determine the functional contribution of RIG-I during bacterial meningitis.

**Methods:**

In this study, we utilized primary murine and immortalized human glial cells to investigate the contribution of RIG-I-mediated responses during *N. meningitidis* and *S. pneumoniae* infection. We used immunoblot analysis and specific-capture ELISAs to quantify changes in RIG-I, interferon-stimulated genes, and type I IFN production. To assess the functional role of RIG-I during bacterial infection of glial cells, we employed both siRNA-mediated knockdown and pharmacological inhibition of RIG-I and downstream signaling, respectively.

**Results:**

We demonstrate that RIG-I is constitutively expressed in human and murine glial cells and is further upregulated upon bacterial infection, with protein levels varying according to both the bacterial agent and glial cell subtype. Importantly, we show that RIG-I contributes to protective type I IFN responses by glial cells, leading to the restriction of bacterial burden. Additionally, our findings suggest that type I IFN signaling via IFNAR and the resulting induction of ISGs are critical for limiting bacterial survival in glial cells. Excitingly, we have also demonstrated that we can employ RIG-I nucleic acid agonists to augment these protective responses in infected glial cells.

**Discussion:**

Our findings establish RIG-I as a key cytosolic sensor that contributes to type I IFN responses in glial cells during bacterial infection of the CNS. By promoting IFNAR-dependent ISG induction, RIG-I signaling contributes to the restriction of bacterial burden. Moreover, our ability to enhance these protective responses using RIG-I agonists highlights the therapeutic potential of targeting this pathway to promote pathogen control during bacterial meningitis.

## 1 Introduction

Bacterial meningitis is a severe and life-threatening infection within the central nervous system (CNS), marked by inflammation of the meninges, and can rapidly progress to cause many neurological complications and mortality if left untreated (1–3). Two causative agents of bacterial meningitis include *Neisseria meningitidis* and *Streptococcus pneumoniae*, both of which are associated with high morbidity and mortality worldwide despite advances in antimicrobial therapies and vaccination strategies (4, 5). *N. meningitidis* and *S. pneumoniae* invade the CNS following dissemination through the bloodstream. While *N. meninigitidis* and *S. pneumoniae* are primarily considered to be extracellular CNS pathogens, a percentage of the bacteria are internalized by resident host cells via distinct bacterial surface adhesins and invasins (6–9). In response to bacterial invasion of the CNS, resident glial cells become activated and initiate a robust immune response characterized by the production of inflammatory mediators, including cytokines and chemokines (10, 11). While this response is essential for the recruitment of peripheral immune cells and pathogen clearance, it also contributes to significant collateral damage, including neuronal injury and blood-brain barrier disruption, which can lead to long-term neurological damage (12, 13).

The resident glial cells, microglia and astrocytes, play a vital role during acute CNS infections such as bacterial meningitis, where they act as the first line of defense by detecting and responding to invading pathogens (14, 15). Microglia, the primary innate immune cells of the CNS, continuously survey the microenvironment for signs of infection or damage and respond through phagocytosis, antigen presentation, and the release of inflammatory mediators (16–18). Astrocytes, though traditionally known for maintaining neuronal homeostasis and supporting BBB integrity, also possess key immunological roles, including cytokine and chemokine production as well as regulation of leukocyte trafficking across the BBB (19, 20). Importantly, both microglia and astrocytes express a range of pattern recognition receptors (PRRs), including Toll-like receptors (TLRs), nucleotide-binding oligomerization domain (NOD)-like receptors (NLRs) and retinoic acid-inducible gene I-like receptors (RLRs), enabling them to detect both extracellular and intracellular pathogen-associated molecular patterns (PAMPs) (21–23). The capacity to sense microbial motifs allows glial cells to initiate and shape the innate immune response during CNS infections. Notably, resident brain cell immune responses can be impacted by the distinct mechanisms of *N. meningitidis* and *S. pneumonaie* pathogenesis including bacterial adhesin, internalization, and production of specific bacterial ligands for PRRs (6, 8, 12, 24).

While extracellular PRRs such as TLRs have been widely characterized in the context of bacterial meningitis, recent evidence suggests that cytosolic nucleic acid sensors may also play a pivotal role in mediating immune responses within the CNS (25–28). Retinoic acid-inducible gene I (RIG-I) is a DExD/H box RNA helicase. Significant structural biology studies have been conducted to characterize this member of the RLR family (29–32). This RNA sensor preferentially recognizes cytosolic RNA with 5'-triphosphate ends via its carboxy-terminal domain (CTD). However, it can additionally recognize single-stranded RNAs or single-stranded RNA hairpins (29, 32–35). Upon ligand binding, RIG-I undergoes a conformational change that exposes the CARD domains and initiates a downstream signaling cascade leading to the production of type I IFNs and proinflammatory cytokines (30, 31, 36–39). Notably, type I IFNs including IFN-β can then signal in a paracrine and autocrine manner via the interferon-alpha/beta receptor (IFNAR), leading to protein production of interferon-stimulated genes (ISGs) that play an important role in innate immune defense. While RIG-I activation of innate immune responses has been extensively studied in antiviral and parasitic immunity, emerging data suggest it may also participate in host responses to bacterial pathogens (32, 40–44). Additionally, RIG-I has been shown to identify bacterial nucleic acids in peripheral cell types (45–47). Notably, our group has previously shown that bacterial nucleic acids derived from *Neisseria meningitidis* and *Streptococcus pneumoniae* can activate RIG-I signaling and result in type I IFN production by glial cells (48). However, the specific contribution of RIG-I to bacterial sensing in the CNS remains poorly defined in regard to its functional role in shaping protective glial cell immune responses.

Here, we demonstrate that RIG-I plays a critical and previously underappreciated role in mediating protective glial cell responses during bacterial CNS infections. We show that RIG-I is differentially activated in response to the bacterial pathogens *Neisseria meningitidis* and *Streptococcus pneumoniae*. Importantly, this study highlights that RIG-I activation drives type I interferon production and ISG induction, both of which contribute to the restriction of bacterial burden. Furthermore, we demonstrate that stimulation with RIG-I agonists enhances restriction of bacterial burden, highlighting the therapeutic potential of targeting this pathway. Together, our findings establish RIG-I as a key initiator of glial cell responses to bacterial infection and highlight it as a potential target to enhance protective immune responses in the CNS.

## 2 Materials and methods

### 2.1 Source and culture of human glial cell line

The human microglia cell line (hμglia) was kindly provided by Dr. Jonathan Karn (Case Western Reserve University). This cell line was established from primary human cells transformed using lentiviral vectors encoding SV40 T antigen and human telomerase reverse transcriptase. Detailed characterization and classification of the hμglia cells have been previously reported (48–51). They exhibit microglia-like morphology as demonstrated by the protein production of surface markers including CD11B, TGFBR, and P2RY12, as well as their phagocytic activity. Cells were cultured in Dulbecco’s Modified Eagle Medium (DMEM) supplemented with 5% fetal bovine serum (FBS) and 100 U/ml penicillin-100 ug/ml streptomycin and maintained at 37°C in 5% CO2 as previously described (34, 42).

### 2.2 Isolation and culture of primary murine glial cells

Primary murine glial cells were isolated as previously described by our laboratory (34, 38–42). The resulting cell suspension was pelleted and resuspended in RPMI 1640 supplemented with 10% FBS and penicillin-streptomycin and cultured for two weeks. Astrocytes were isolated from these mixed glial cultures by treatment with 0.25% trypsin and 1mM EDTA and maintained in RPMI 1640 with 10% FBS and penicillin-streptomycin. Microglia were maintained in RPMI 1640 containing 10% FBS and 20% conditioned media from LADMAC cells (ATCC, Cat #CRL-2420, RRID: CVCL_2550), a murine monocyte-like cell line that secretes colony-stimulating factor 1 (CSF-1), required for microglial viability.

### 2.3 Bacterial propagation

*Neisseria meningitidis* strain MC58 (ATCC BAA-335) and *Streptococcus pneumoniae* strain CS109 (ATCC 51915) were grown on Columbia agar plates supplemented with 5% defibrinated sheep blood (BD 221263) and cultured in Columbia broth (BD 294420) at 37°C and 5% CO_2_ overnight. The number of colony-forming units (CFUs) for each bacterial species was determined using a Genespec3 spectrophotometer as previously described (MiraiBio Inc.) (48, 52).

### 2.4 Bacterial infection of glial cells

Glial cells were seeded at a density of 5 x 10^4^ cells per well and infected with *N. meningitidis* or *S. pneumoniae* at varying multiplicities of infection (MOIs) in antibiotic-free media for 2 hours as previously described (48). Following infection, the medium was replaced with fresh medium containing 1% penicillin-streptomycin. At the designated time points, cell supernatants and whole-cell protein lysates were collected for analysis.

### 2.5 siRNA transfection of glial cells

Glial cells were transfected using RNAiMAX following the manufacturer’s guidelines and as previously described (48). Silencer Select small interfering RNAs (siRNAs; Thermo Fisher Scientific) were used at a final concentration of 5nM (human microglia) or 10nM (murine microglia and astrocytes). These included siRNAs targeting RIG-I (assay ID: s223615 (human), s106376 and s106374 (murine)], or a scrambled negative control siRNA [catalog number: AM4611 (human) and AM4613 (murine)]. Transfection with Silencer Select siRNAs was performed 24 hours prior to infection with *N. meningitidis* or *S. pneumoniae*. Cell supernatants and whole-cell protein lysates were collected at the specified time points. Viable *N. meningitidis* and *S. pneumoniae* CFUs were plated at the specified time points.

### 2.6 IFN-β treatment of glial cells

Glial cells were exogenously treated with human (R&D Systems; 8999-IF) or murine (VWR; 10762-364) recombinant IFN-β at a final concentration of 1ng/mL in antibiotic-free medium for 2 hours prior to infection by *N. meningitidis* or *S. pneumoniae* as previously described. Cell supernatants and whole-cell protein lysates were collected at the indicated time points. Viable *N. meningitidis* and *S. pneumoniae* CFUs were plated at the specified time points.

### 2.7 Ligand transfection of glial cells

Pattern recognition receptor (PRR) ligands including B-DNA [poly(dA:dT); VWR; 1μg/mL], 5’-triphosphate double-stranded RNA (5’ppp dsRNA; InvivoGen; 5μg/mL), and polyinosinic:polycytidylic acid (polyI:C; InvivoGen; 1μg/mL) were incubated with the lipid-based transfection reagent, Lipofectamine 2000 (L2K; Invitrogen; 7μg/mL) for 15 minutes in reduced-serum medium. Additionally, glial cells were treated exogenously with lipopolysaccharide (LPS; Cell Signaling 10ng/mL). The resulting reactions were applied to glial cells and incubated for 2 hours. Medium was replaced with fresh medium in the absence of transfection reagents and cells were then infected with *N. meningitidis* or *S. pneumoniae* as previously described. At designated time points, cell supernatants and whole-cell protein lysates were collected and viable *N. meningitidis* and *S. pneumoniae* CFUs were plated.

### 2.8 Inhibitor treatment of glial cells

Glial cells were exogenously treated with the TBK1/IKKϵ inhibitor, BX795 (5μg/mL), or the STAT1 inhibitor, Fludarabine (10nM) in antibiotic-free medium for 2 hours prior to infection by *N. meningitidis* or *S. pneumoniae* as previously described. Cell supernatants and whole-cell protein lysates were collected at the indicated time points. Viable *N. meningitidis* and *S. pneumoniae* CFUs were plated at the specified time points.

### 2.9 Enzyme-linked immunosorbent assays

Specific capture enzyme-linked immunosorbent assays (ELISAs) were conducted to measure the levels of immune mediator production in response to ligands, *N. meningitidis*, or *S. pneumoniae*. Concentrations of IL-6 [BD Biosciences; Cat #554543, #554546, RRID: AB_398568, AB_395470 (human), BD Biosciences; Cat #554400, #554402, RRID: AB_398549, AB_395368) (murine)] and IFN-β [R&D Systems; DY814-05 (human), BD Biosciences; 519202, 508105 (murine)] were determined using commercially available antibody pairs or ELISA kits following established protocols (53, 54). Standard curves were generated using recombinant proteins for each target. Protein concentrations in cell supernatants were calculated by extrapolating sample absorbance values to the corresponding standard curve.

### 2.10 Immunoblot analysis

Whole-cell protein lysates were analyzed by chemiluminescent immunoblotting to assess the expression levels of various proteins, including RIG-I, IFIT-1, and IFIT-3. Detection was performed using a rabbit monoclonal antibody against RIG-I (Cell Signaling; 3743S), a rabbit monoclonal antibody against IFIT-1 [Cell Signaling; 14769S (human)], a murine monoclonal antibody against IFIT-1 [Novus Biologicals; NBP2-02340 (murine)], a rabbit monoclonal antibody against IFIT-3 [Cell Signaling; 47676 (human)], and a murine monoclonal antibody against IFIT-3 [Novus Biologicals; NBP2-02148 (murine)]. To ensure equal protein loading, blots were also probed with a rabbit monoclonal antibody against β-actin (Cell Signaling; 4957S). Densitometric quantification was performed using Azure Spot Pro (Azure Biosystems), and the protein levels of RIG-I, IFIT-1, and IFIT-3 were normalized to β-actin expression.

### 2.11 Statistical analysis

Data are expressed as the mean ± standard error of the mean (SEM). Statistical analyses were performed using GraphPad Prism (GraphPad Software, La Jolla, CA, USA). According to the experimental design, comparisons were made using Student’s *t*-test, one-way analysis of variance (ANOVA) followed by Dunnett’s *post hoc* test, or two-way ANOVA with Šídák’s multiple comparisons test as appropriate. A P-value of less than 0.05 was considered indicative of statistical significance.

## 3 Results

### 3.1 Glial cells produce RIG-I and release IFN-β in response to bacterial infection with *N. meningitidis* and *S. pneumoniae*.

**Figure 1:**
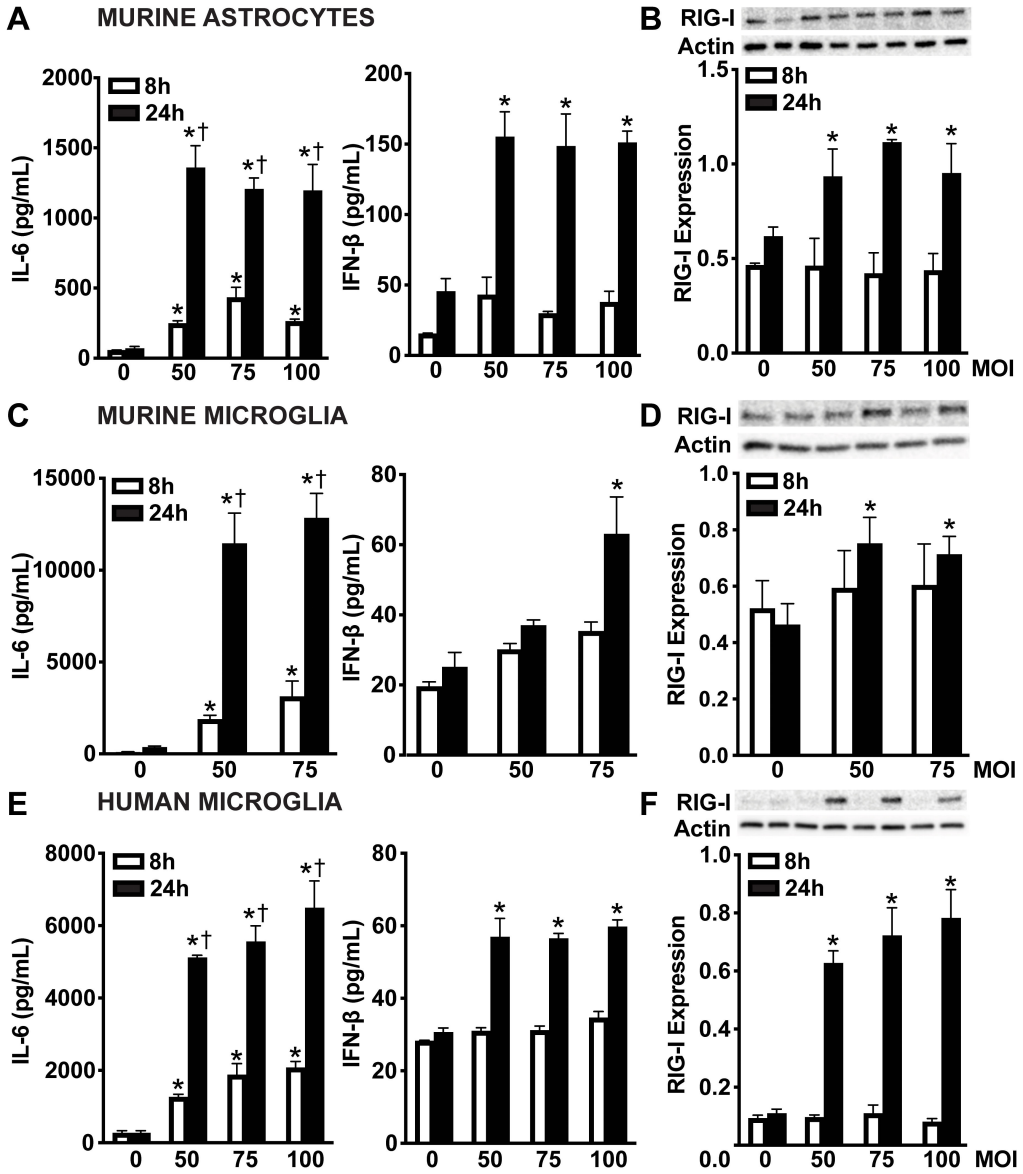
Glial cells release the type I IFN, IFN-β and upregulate RIG-I following *N. meningitidis* challenge. Primary murine astrocytes **(A, B)**, primary murine microglia **(C, D)**, and human microglia **(E, F)** were uninfected (0) or infected with *N. meningitidis* at MOIs of 50:1, 588 75:1, or 100:1. Panels **(A, C, E)**: At 8 and 24 h post-infection, IL-6 and IFN-β production was 589 assessed by specific capture ELISAs in primary murine astrocytes, primary murine microglia, and human microglia, respectively. Asterisks indicate a statistically significant difference compared to uninfected cells. Daggers indicate a statistically significant difference compared to similarly challenged cells at 8 h post-infection. Panels **B**, **D**, and **F**: Protein production of RIG-I (102 kDa) was assessed at 8 and 24 h post-infection by immunoblot analysis in primary murine astrocytes, primary murine microglia, and human microglia, respectively. Representative immunoblots and the average protein production of RIG-I quantified by densitometric analysis and normalized to β-actin levels are shown. Asterisks indicate a statistically significant difference compared to uninfected cells (Mean ± SEM, n=3; two-way ANOVA, P value < 0.05).

Our previous data revealed an underappreciated role for cytosolic nucleic acid sensors in glial responses to bacterial components from *N. meningitidis* and *S. pneumoniae*, notably showing increased RIG-I protein production following stimulation with flagellin, lipopeptides, lipopolysaccharide, as well as with bacterial DNA and RNA (48). To further investigate the role of cytosolic nucleic acid sensors in glial recognition of viable *N. meningitidis* and *S. pneumoniae*, we evaluated both RIG-I protein production and downstream cytokine responses. Upon challenge with *N. meningitidis*, primary murine astrocytes (**Figure 1A**), primary murine microglia (**Figure 1C**) and human microglia (**Figure 1E**) exhibited a time-dependent production of the proinflammatory cytokine, interleukin-6 (IL-6). Notably, IFN-β release was increased at 24 hours post-infection, but not at 8 hours post-infection, indicating a delayed type I IFN response. Among glial cell populations, primary murine astrocytes (**Figure 1B** and **Supplementary Figure S1A**) and microglia (**Figure 1D** and **Supplementary Figure S1B**) demonstrated constitutive RIG-I protein production, whereas human microglia (**Figure 1F** and **Supplementary Figure S1C**) displayed lower constitutive levels. However, RIG-I protein was markedly elevated in all cell types following *N. meningitidis* challenge, coinciding with enhanced cytokine production.

**Figure 2:**
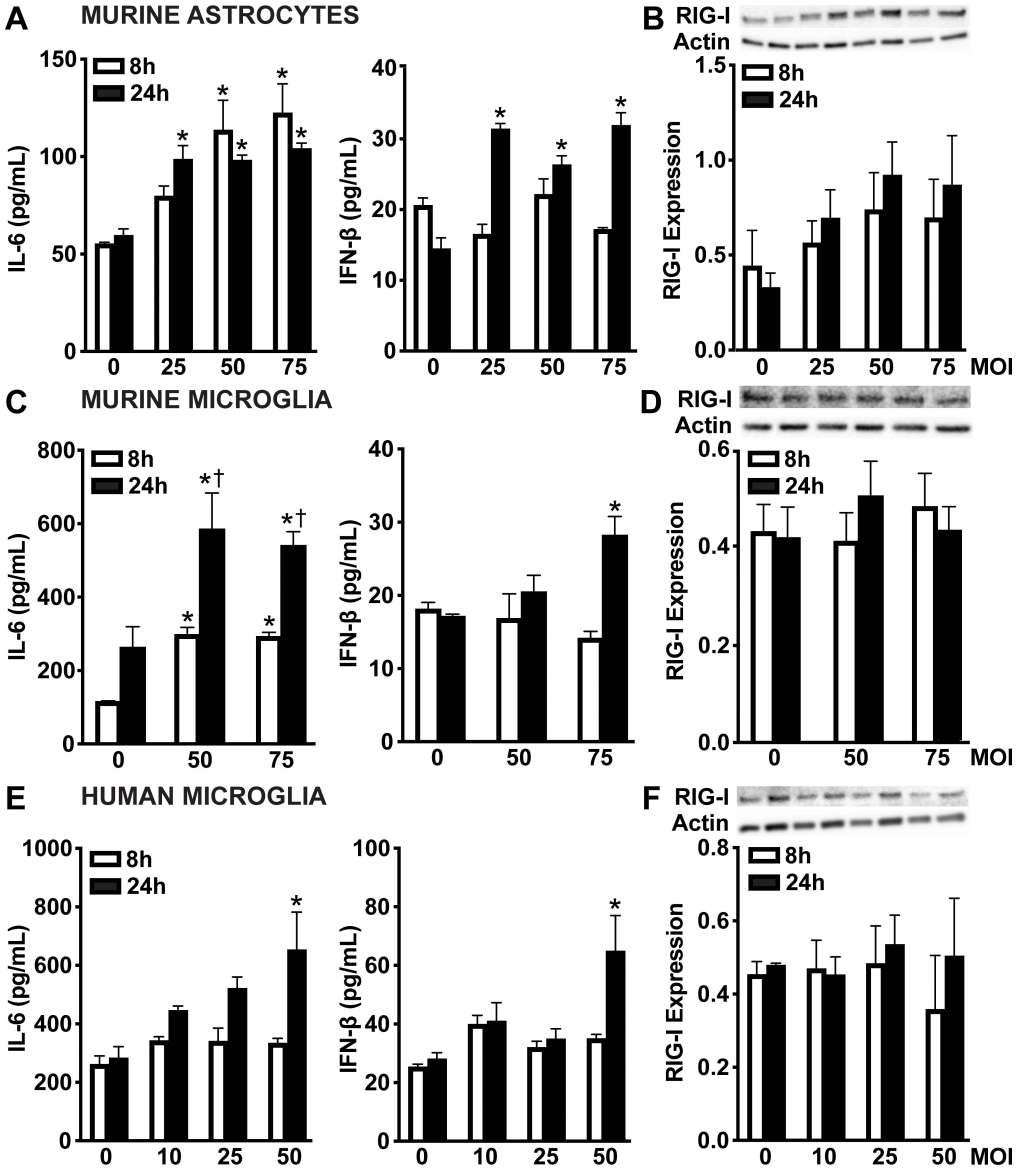
Glial cells release IFN-β and demonstrate constitutive protein levels of RIG-I following *S. pneumoniae* challenge. Primary murine astrocytes **(A, B)**, primary murine microglia **(C, D)**, and human microglia **(E, F)** were uninfected (0) or infected with *S. pneumoniae* at MOIs of 10:1, 25:1, 50:1 or 75:1. Panels **(A, C, E)**: At 8 and 24 h post-infection, IL-6 and IFN-β production was assessed by specific capture ELISAs in primary murine astrocytes, primary murine microglia, and human microglia, respectively. Asterisks indicate a statistically significant difference compared to uninfected cells. Daggers indicate a statistically significant difference compared to similarly challenged cells at 8 h post-infection. Panels **B**, **D**, and **F**: Protein production of RIG-I (102 kDa) was assessed at 8 and 24 h post-infection by immunoblot analysis in primary murine astrocytes, primary murine microglia, and human microglia, respectively. Representative immunoblots and the average protein levels of RIG-I quantified by densitometric analysis and normalized to β-actin levels are shown. Asterisks indicate a statistically significant difference compared to uninfected cells (Mean ± SEM, n=3; two-way ANOVA, P value < 0.05).

Similarly, primary murine astrocytes (**Figure 2A**), primary murine microglia (**Figure 2C**), and human microglia (**Figure 2E**) showed time-dependent IL-6 production following *S. pneumoniae* infection. However, all glial cell types displayed lower levels of IFN-β production during *S. pneumoniae* infection compared to those observed during *N. meningitidis* infection. While murine astrocytes released increased IFN-β across all MOIs, murine and human microglia produced increased IFN-β only at the highest MOI. Notably, in contrast to the robust RIG-I upregulation observed with *N. meningitidis*, RIG-I protein levels remained unchanged across both murine and human microglia following *S. pneumoniae* challenge (**Figures 2B, D, F** and **Supplementary Figures S2A-C**). While not statistically significant, murine astrocytes exhibited a trend toward RIG-I upregulation during *S. pneumoniae* infection (**Figure 2B** and **Supplementary Figure S2A**). Collectively, these findings indicate that glial cells mount distinct responses to *N. meningitidis* and *S. pneumoniae* infections, characterized by differential induction of RIG-I, IL-6, and type I IFN production. These results further suggest a potential role for RIG-I in pathogen recognition and modulation of both inflammatory and type I IFN responses.

### 3.2 *N. meningitidis* and *S. pneumoniae* challenge stimulate RIG-I-dependent production of IFN-β in glial cells

**Figure 3:**
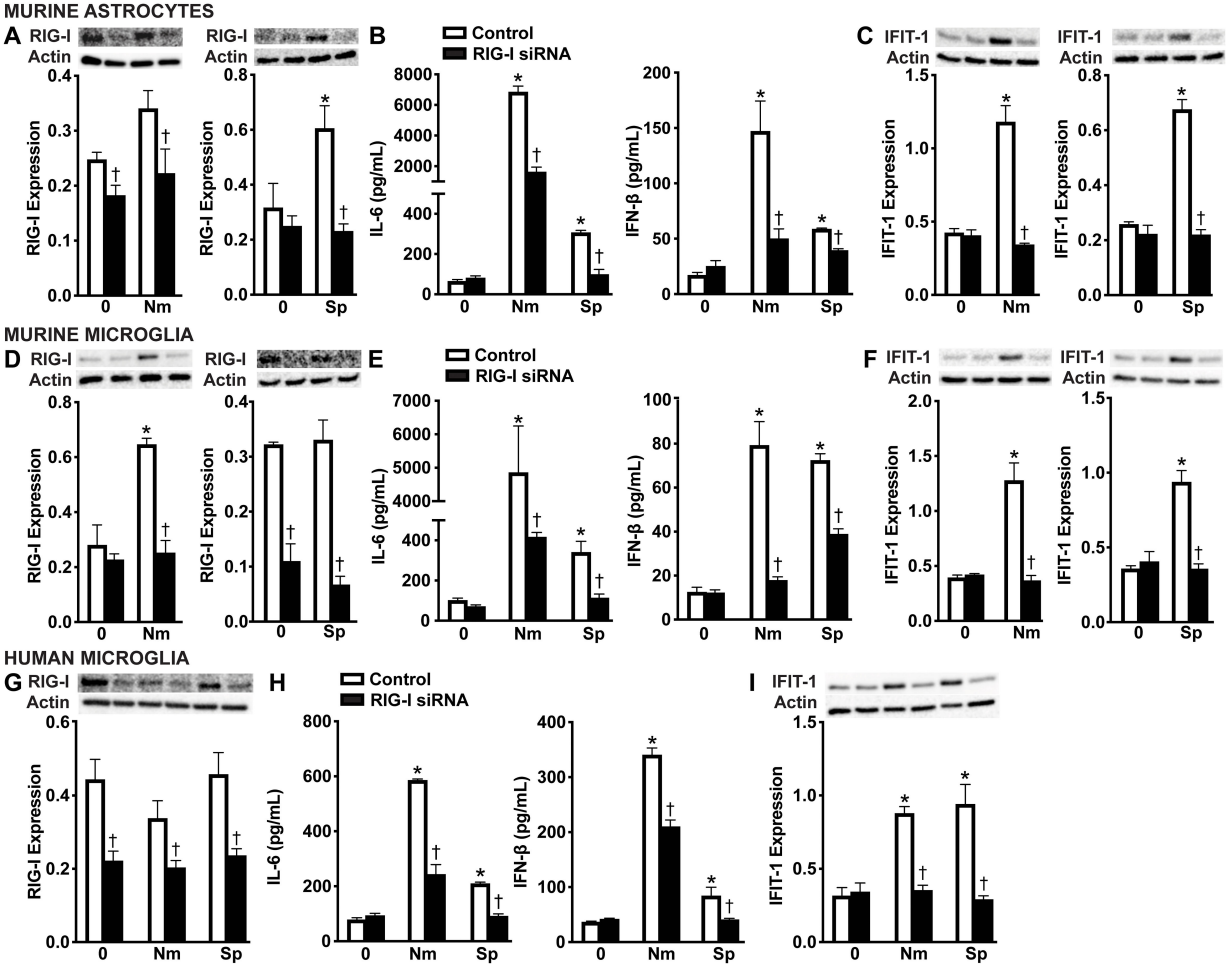
RIG-I mediates glial cell type I IFN responses to *N. meningitidis* and *S. pneumoniae* challenge. Primary murine astrocytes **(A-C)**, primary murine microglia **(D-F)**, and human microglia **(G-I)** were transfected with siRNA (5 nM or 10 nM) directed against RIG-I or control siRNA using RNAiMAX. Cells were then left uninfected (0) or challenged with *N. meningitidis* (Nm) or *S. pneumoniae* (Sp) (MOI of 75:1). At 24 h post-infection, protein production of RIG-I (102 kDa) and IFIT-1 (56 kDa) was assessed by immunoblot analysis in primary murine astrocytes **(A, C)**, primary murine microglia **(D, F)**, and human microglia **(G, I)** respectively. Representative immunoblots and the average protein levels of RIG-I and IFIT-1 quantified by densitometric analysis and normalized to β-actin levels are shown. Additionally, IL-6 and IFN-β production was assessed by specific capture ELISAs in primary murine astrocytes **(B)**, primary murine microglia **(E)**, and human microglia **(H)**. Asterisks indicate a statistically significant difference compared to uninfected cells. Daggers represent a significant reduction compared to the infected control siRNA-treated group (Mean ± SEM, n=3; two-way ANOVA, P value < 0.05).

In order to determine if RIG-I contributes to type I IFN production by glial cells following bacterial infection, we employed an siRNA approach to knockdown the expression of RIG-I (**Figures 3A, D, G**, and **Supplementary Figures S3A-E**). As shown in **Figure 3**, we observed significant knockdown of RIG-I protein production in primary murine astrocytes, primary murine microglia, and human microglia compared to the control-treated group (**Figures 3A, D, G** and **Supplementary Figures S3A-E**). As expected, infection with both pathogens resulted in robust IL-6 and significant IFN-β production by all glial populations compared to the cells treated with the negative-control siRNA (**Figures 3B, E, H**). Notably, RIG-I knockdown significantly reduced IL-6 and IFN-β production across all cell types, indicating that RIG-I plays a role in stimulating both inflammatory and type I IFN responses during bacterial infection (**Figures 3B, E, H**). Consistent with decreased IFN-β production, knockdown of RIG-I also led to a marked reduction in expression of the ISG, IFIT-1 in all cell types (**Figures 3C, F, I**F and **Supplementary Figures S3F-J**), further supporting its role in driving downstream type I IFN signaling.

### 3.3 Interferon-stimulated genes are expressed in glial cells following *N. meningitidis* and *S. pneumoniae* challenge

**Figure 4:**
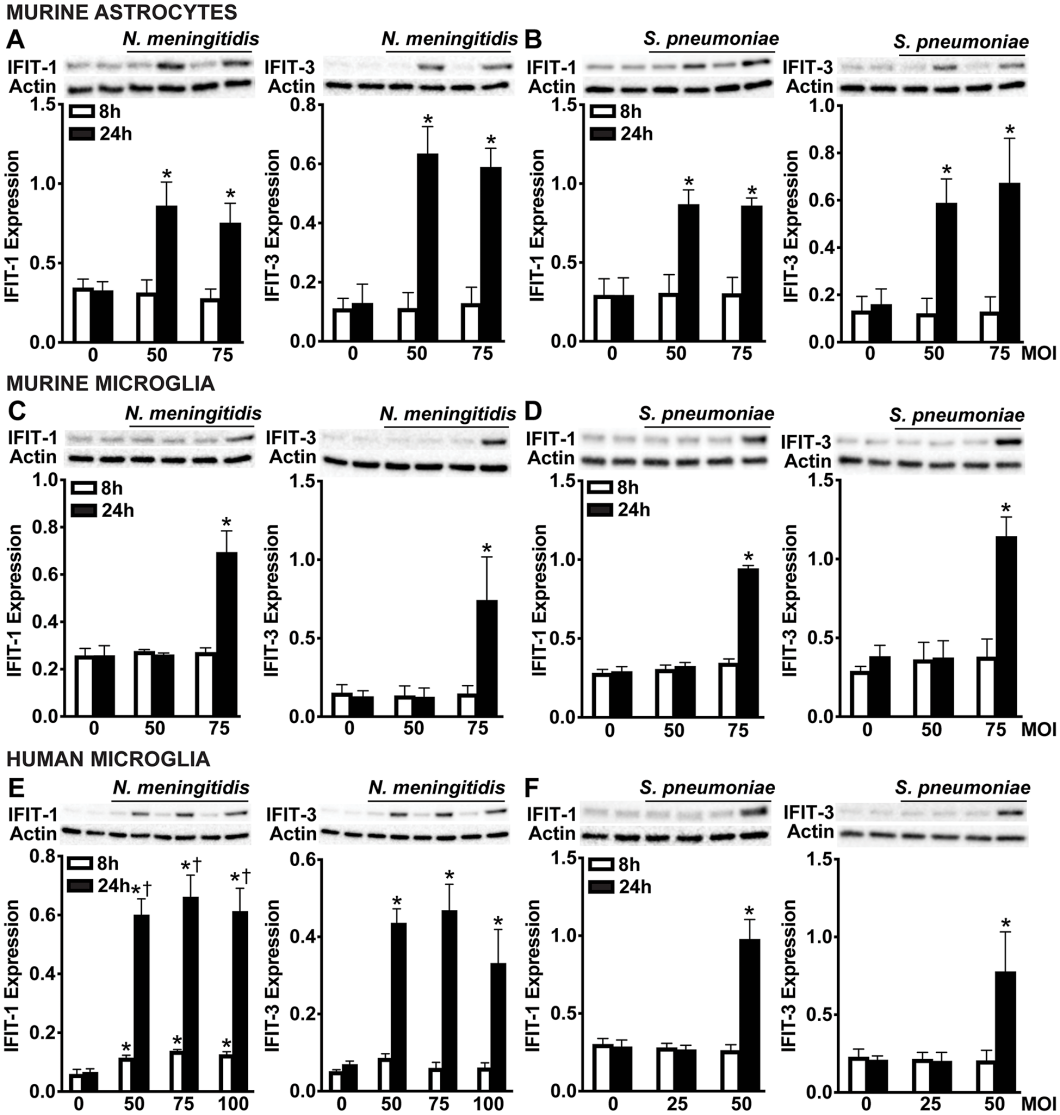
Glial cells upregulate ISGs, IFIT-1 and IFIT-3 following *N. meningitidis* and *S. pneumoniae* challenge. Primary murine astrocytes **(A, B)**, primary murine microglia **(C, D)**, and human microglia **(E, F)** were left uninfected (0) or challenged with *N. meningitidis* (MOI 50:1, 75:1, and 100:1) or *S. pneumoniae* (MOI 25:1, 50:1, and 75:1). At 24 h post-infection, protein production of IFIT-1 (56 kDa) and IFIT-3 (60-65 kDa) was assessed by immunoblot analysis. Representative immunoblots and the average protein levels of IFIT-1 and IFIT-3 quantified by densitometric analysis and normalized to β-actin levels are shown. Asterisks indicate a statistically significant difference compared to uninfected cells. Daggers indicate a statistically significant difference compared to challenged cells at 8 h post-infection (Mean ± SEM, n=3; two-way ANOVA, P value < 0.05).

Type I IFNs are known to induce the expression and protein production of ISGs via paracrine and autocrine activation of IFNAR (55, 56). To assess whether glial cell production of IFN-β during bacterial infection promotes ISG protein production, we measured the levels of two canonical ISGs, including IFN-induced proteins with tetratricopeptide repeats 1 (IFIT-1) and IFIT-3. As shown in **Figure 4**, primary murine astrocytes significantly upregulated both IFIT-1 and IFIT-3 at 24 hours post-infection in response to *N. meningitidis* (**Figure 4A** and **Supplementary Figures S4A, B**) and *S. pneumoniae* (**Figure 4B** and **Supplementary Figures S4C, D**) across all tested MOIs. These findings align with our earlier results demonstrating sustained production of IFN-β during infection (**Figures 1A**, **2A**, **3B, E, H**). In contrast, primary murine microglia exhibited significant induction of IFIT-1 and IFIT-3 only at the highest MOI for both pathogens, corresponding with the elevated IFN-β levels observed under these conditions (**Figures 4C, D** and **Supplementary Figures S4E-H**). Similarly, human microglia displayed robust ISG induction at all MOIs during *N. meningitidis* infection (**Figure 4E** and **Supplementary Figures S4I, J**), but only at the highest MOI in response to *S. pneumoniae* (**Figure 4F** and **Supplementary Figures S4K, L**), again consistent with the observed production of IFN-β by these cells (**Figures 1E**, **2E**). Notably, ISG induction was not observed at earlier time points, suggesting that it is not a direct response to pathogen recognition, but rather a secondary effect mediated by the secretion of IFN-β. This delayed onset supports a model in which IFN-β, once secreted by infected cells, signals through IFNAR in both an autocrine and paracrine manner to drive ISG transcription in glial cells [55, 56].

### 3.4 RIG-I initiates protective type I interferon responses that restrict *N. meningitidis* and *S. pneumoniae* burden in glial cells

**Figure 5:**
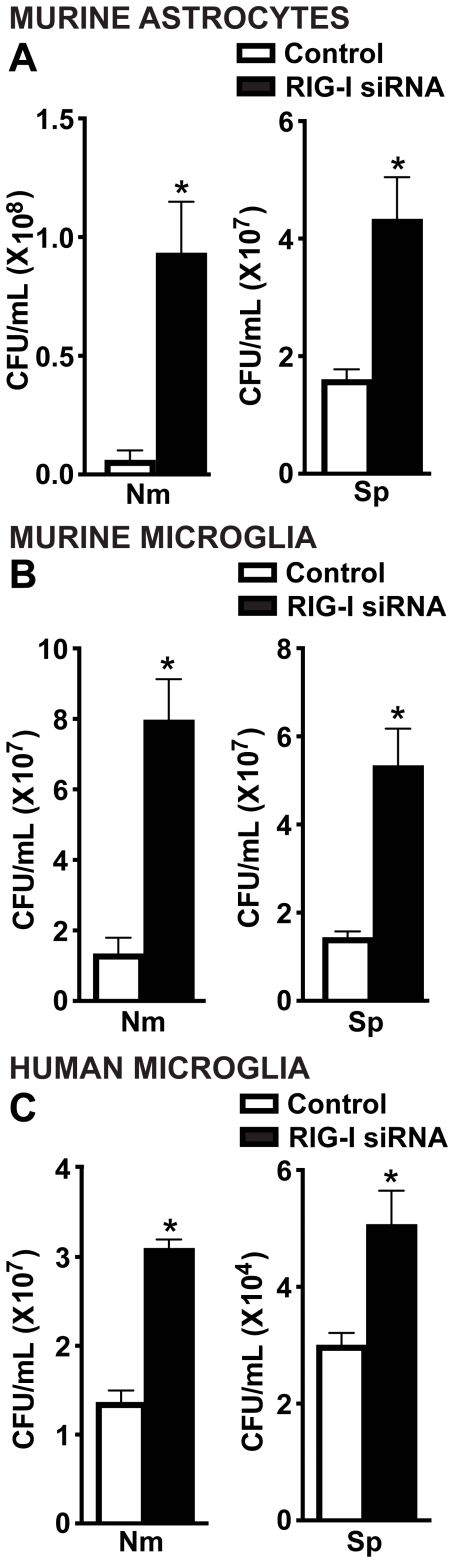
RIG-I mediates antimicrobial responses of glial cells to *N. meningitidis* and *S. pneumoniae* infection. Primary murine astrocytes **(A)**, primary murine microglia **(B)** and human microglia **(C)** were transfected with siRNA (5 nM or 10 nM) directed against RIG-I or control siRNA using RNAiMAX. Cells were then challenged with *N. meningitidis* (Nm) or *S. pneumoniae* (Sp) (MOI of 50:1). At 24 h post-infection, cells were lysed, and viable bacteria were assessed by colony counting. Asterisks represent a significant increase compared to the control siRNA-treated group (Mean ± SEM, n=3; one-way ANOVA, P value < 0.05).

To investigate the role of RIG-I in mediating protective glial cell responses during infection, we assessed bacterial viability of *N. meningitidis* and *S. pneumoniae* following RIG-I knockdown. Excitingly, RIG-I knockdown led to a significant increase in bacterial survival of both *N. meningitidis* and *S. pneumoniae* in primary murine astrocytes (**Figure 5A**), primary murine microglia (**Figure 5B**), and human microglia (**Figure 5C**) relative to control-treated cells. These findings support that RIG-I contributes to the restriction of bacterial burden in infected glial cells.

**Figure 6:**
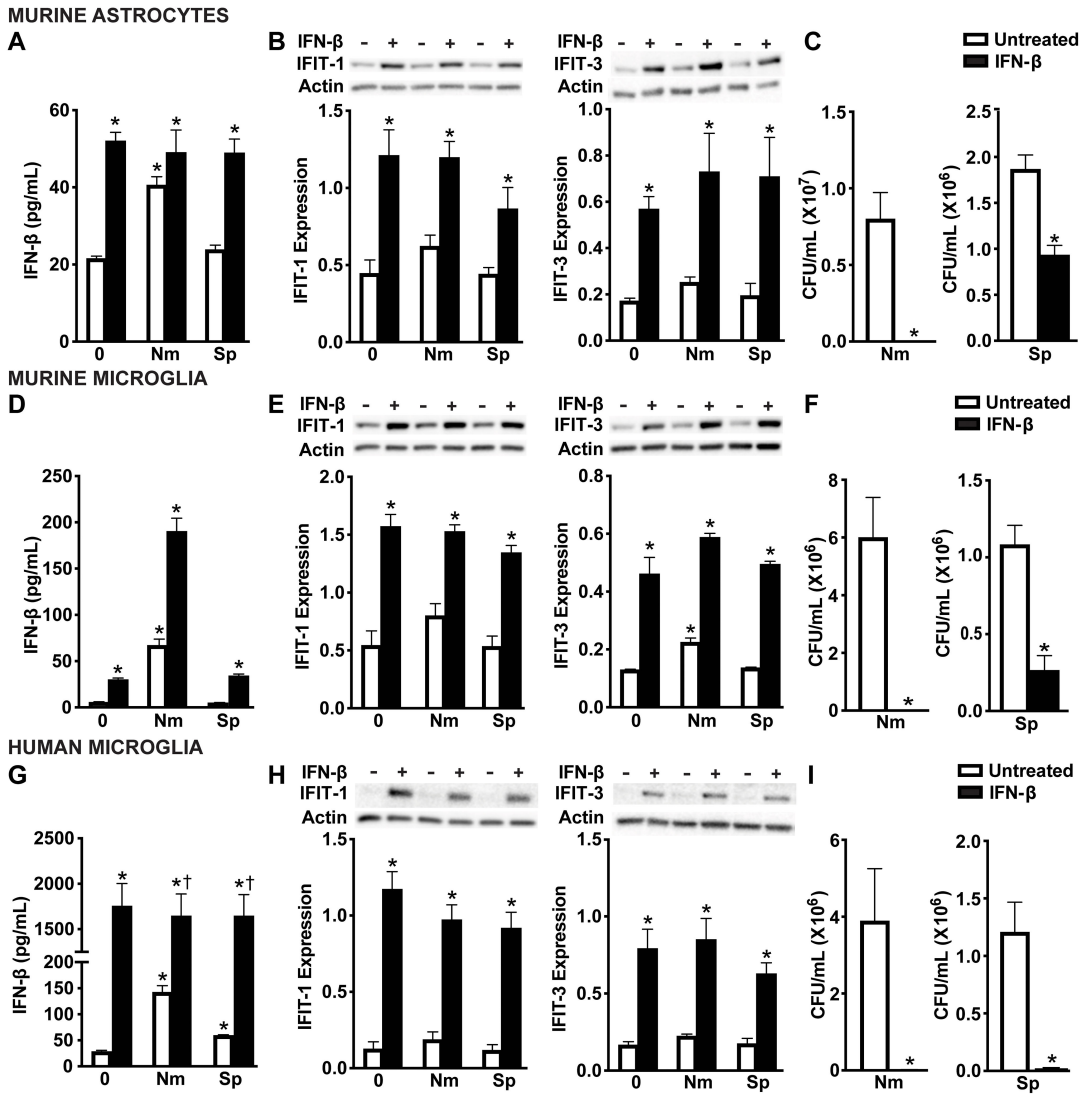
IFN-mediated responses by glial cells restrict *N. meningitidis* and *S. pneumoniae* viability. Primary murine astrocytes **(A-C)**, primary murine microglia **(D-F)**, and human microglia **(G-I)** were left untreated or treated with recombinant IFN-β (1 ng/mL) for 2 h. Cells were then left uninfected (0) or infected with *N. meningitidis* (Nm) or *S. pneumoniae* (Sp) (MOI 50:1). At 6 h post-infection, IFN-β treatment was confirmed through specific-capture ELISAs in primary murine astrocytes **(A)**, primary murine microglia **(D)**, and human microglia **(G)**. Additionally, protein production of IFIT-1 (56 kDa) and IFIT-3 (60-65 kDa) was assessed by immunoblot analysis in primary murine astrocytes **(B)**, primary murine microglia **(E)**, and human microglia **(H)**. Representative immunoblots and the average protein levels of IFIT-1 and IFIT-3 quantified by densitometric analysis and normalized to β-actin levels are shown. Viable *N. meningitidis* and *S. pneumoniae* were assessed by colony counting in primary murine astrocytes **(C)**, primary murine microglia **(F)**, and human microglia **(I)**. Asterisks indicate a statistically significant difference compared to untreated cells. Daggers represent a significant increase compared to the infected and untreated control (Mean ± SEM, n=3-6; two-way ANOVA **(A, B, D, E, G, H)**, or one-way ANOVA **(C, F, I)**, P value < 0.05).

To determine whether these RIG-I-dependent type I IFNs are sufficient to mediate these protective effects, we next treated glial cells with IFN-β prior to infection. As expected, exogenous IFN-β treatment significantly increased detectable IFN-β levels (**Figures 6A, D, G**) and also led to upregulation of IFIT-1 and IFIT-3 across all glial cell types (**Figures 6B, E, H** and **Supplementary Figure S5A-F**). Consistent with RIG-I initiation of protective responses, treatment with IFN-β significantly reduced the viability of both *N. meningitidis* and *S. pneumoniae* in all glial cell types (**Figures 6C, F, I**), reinforcing the protective function of type I IFNs during bacterial infection. Notably, IFN-β treatment was more effective at limiting *N. meningitidis* burden as we were unable to recover countable CFU. In contrast, IFN-β treatment significantly reduced *S. pneumoniae* burden in all glial cell types, however it was to a lesser extent in murine astrocytes and microglia. The reduction in *N. meningitidis* and *S. pneumoniae* burden following exogenous IFN-β treatment additionally supports that IFN-β alone is sufficient to mediate these downs.

**Figure 7:**
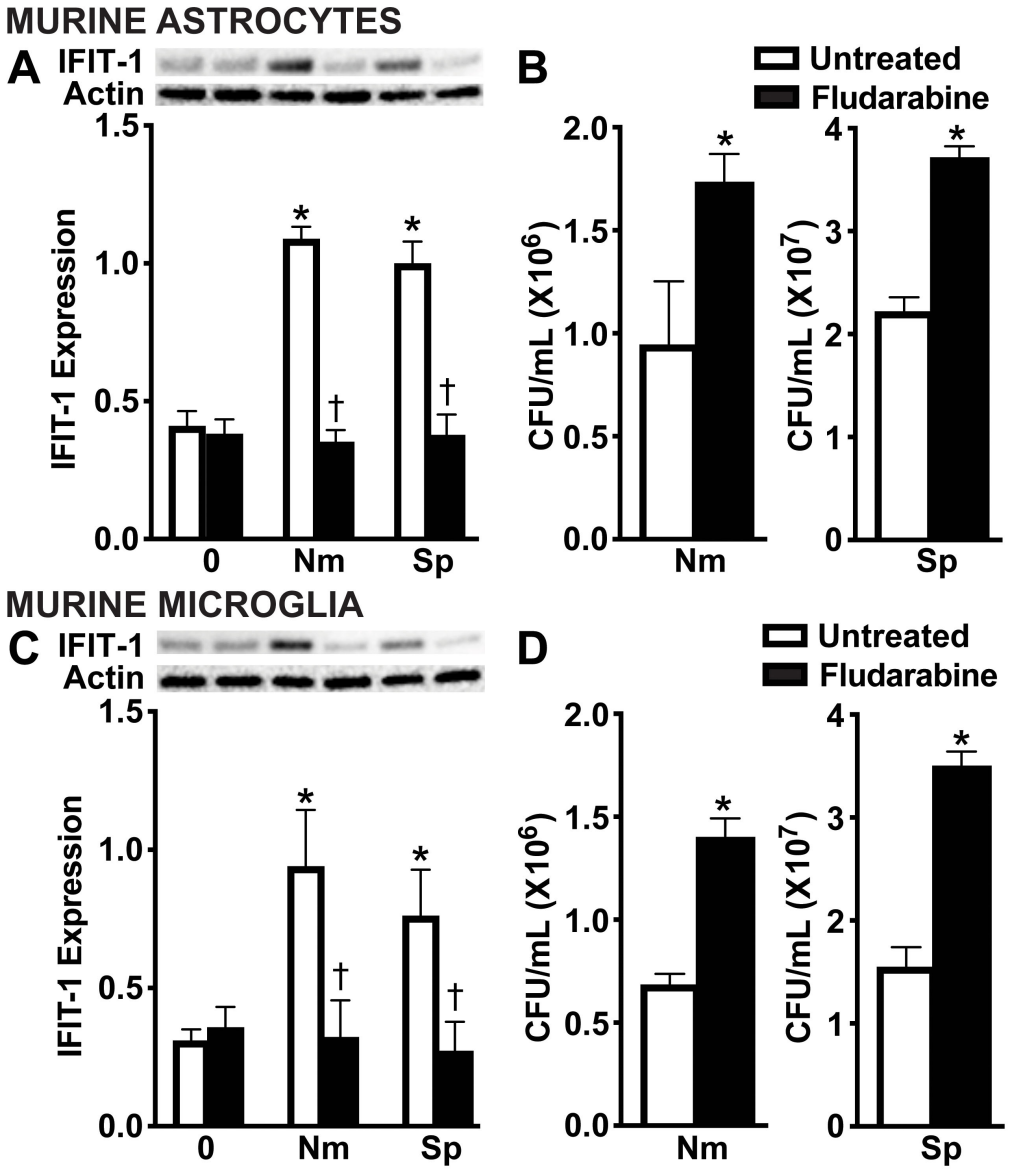
IFNAR downstream signaling is necessary for restriction of bacteria in infected glial cells. Primary murine astrocytes **(A, B)** and primary murine microglia **(C, D)** were left untreated or treated with Fludarabine (10 nM) for 2 h. Cells were uninfected (0) or infected with *N. meningitidis* (Nm) or *S. pneumoniae* (Sp) (MOI 50:1). At 24 h post-infection, protein production of IFIT-1 (56 kDa) was assessed by immunoblot analysis in primary murine astrocytes **(A)** and primary murine microglia **(C)**. Representative immunoblots and the average protein levels of IFIT-1 were quantified by densitometric analysis and normalized to β-actin levels are shown. Asterisks indicate a statistically significant difference compared to uninfected cells. Daggers represent a significant reduction compared to the infected and untreated control. Bacterial burden was assessed by colony counting in primary murine astrocytes **(B)** and primary murine microglia **(D)**. Asterisks indicate a statistically significant difference compared to untreated cells (Mean ± SEM, n=3; two-way ANOVA (A, C) or Student’s t-test **(B, D)**, P value < 0.05).

Type I IFNs signal through the IFNAR in both an autocrine and paracrine manner to induce ISG protein production (55-57)To evaluate the contribution of IFN-β activation of IFNAR to the observed protective responses, we pharmacologically inhibited the downstream signaling protein, STAT1 using Fludarabine (58). As shown in **Figure 7**, Fludarabine treatment significantly reduced the protein production of the ISG, IFIT-1, in both primary murine astrocytes (**Figure 7A** and **Supplementary Figure S6A**) and primary murine microglia (**Figure 7C** and **Supplementary Figure S6B**), consistent with impaired STAT1 signaling. Notably, inhibition of this pathway also resulted in a significant increase in bacterial survival of both *N. meningitidis* and *S. pneumoniae* in primary murine astrocytes (**Figure 7B**) and primary murine microglia (**Figure 7D**), suggesting a loss of the protective effects mediated by type I IFN signaling. Together, these findings demonstrate that type I IFN signaling through IFNAR and subsequent ISG induction is essential for restricting bacterial survival in glial cells.

### 3.5 RIG-I agonists drive protective glial type I interferon responses that restrict bacterial survival

**Figure 8:**
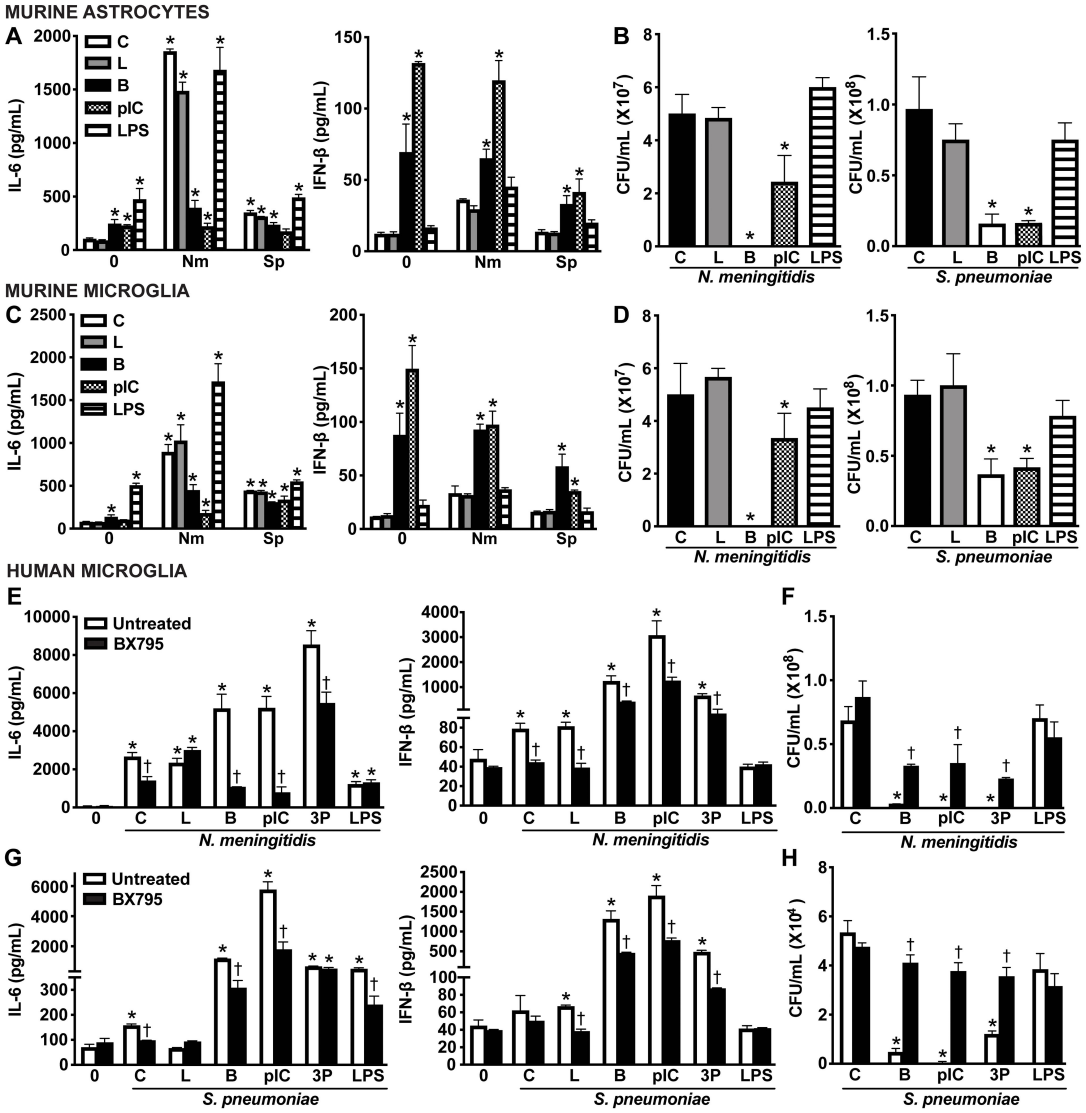
Intracellular nucleic acid delivery elicits protective IFN responses by glial cells. Primary murine astrocytes **(A, B)** and primary murine microglia **(C, D)** were untreated (Control, C) or transfected with ligands including B-DNA (B; 1 μg/mL) and polyinosinic-polycytidylic acid (pIC; 1 μg/mL) using Lipofectamine 2000 (L), or exogenously treated with lipopolysaccharide (LPS; 10 ng/mL) for 2 h. Cells were left uninfected (0) or infected with *N. meningitidis* or *S. pneumoniae* (MOI 50:1). At 8 h post-infection, IL-6 and IFN-β production was assessed by specific capture ELISAs in primary murine astrocytes **(A)** and primary murine microglia **(C)**. Bacterial burden was assessed in primary murine astrocytes **(B)** and primary murine microglia **(D)** by colony counting. Furthermore, human microglia were left untreated or treated with a TBK1/IKKε inhibitor (BX795; 5 ug/mL) for 2 h prior to stimulation with ligands including B-DNA (B; 1 μg/mL), polyinosinic-polycytidylic acid (pIC; 1 ug/mL), and 5’-triphosphosphate double-stranded RNA (3P; 5 μg/mL) using Lipofectamine 2000 **(L)**, or exogenously treated with lipopolysaccharide (LPS; 10 ng/mL) for 2 h. Cells were uninfected (0) or infected with *N. meningitidis* or *S. pneumoniae* (MOI 50:1). At 8 h post-infection, IL-6 and IFN-β production was assessed by specific capture ELISAs following infection with *N. meningitidis***(E)** or *S. pneumoniae***(G)**. Additionally, viable *N. meningitidis***(F)** and *S. pneumoniae***(H)** were assessed by colony counting. Asterisks indicate a statistically significant difference compared to uninfected or untreated control cells. Daggers represent a significant reduction compared to similarly challenged untreated controls (Mean ± SEM, n=3; two-way ANOVA, P value < 0.05).

To further define the functional role of RIG-I in glial cells, we employed well-characterized nucleic acid ligands for RIG-I. Glial cells were challenged with intracellular delivery of non-specific DNA and RNA RIG-I agonists, including B-DNA and polyI:C, as well as the RIG-I-specific ligand, 5’ppp RNA. As shown in **Figure 8**, both primary murine astrocytes (**Figure 8A**) and primary murine microglia (**Figure 8C**) produced significant levels of IL-6 and elicited a strong type I IFN response in response to known ligands (**Figures 8A, C**). Excitingly, treatment with nucleic acid ligands significantly reduced the viability of both *N. meningitidis* and *S. pneumoniae* in primary murine astrocytes (**Figure 8B**) and primary murine microglia (**Figure 8D**), indicating ligand-enhanced restriction of bacterial survival. Consistent with the absence of type I IFN production, LPS did not lead to further reduction in bacterial burden. We observed similar trends in IL-6 and IFN-β production by human microglia in response to nucleic acid ligands and in the presence of either *N. meningitidis* or *S. pneumoniae* infection (**Figures 8E, G** and **Supplementary Figure S7**). Consistent with the observed data in the primary murine glial cells, stimulation with nucleic acid ligands led to a dramatic reduction in *N. meningitidis* and *S. pneumoniae* burden in the human microglia (**Figures 8F, H**). To attribute the observed protective effects to RIG-I activation and initiation of innate immune responses, we employed BX795, the pharmacological inhibitor of the downstream signaling proteins, TBK1/IKKɛ (59). Treatment with BX795 significantly reduced IFN-β production in response to nucleic acid ligands (**Figures 8E, G**) and resulted in a corresponding increase in bacterial burden, compared to untreated controls (**Figures 8F, H**). Together, these findings demonstrate that activation of cytosolic nucleic acid sensors, including RIG-I, with known agonists stimulate enhanced type I IFN responses and restriction of bacterial burden in glial cells, highlighting the potential to therapeutically employ such agonists during bacterial meningitis.

## 4 Discussion

Bacterial meningitis remains a major cause of neurological morbidity and mortality worldwide, driven in part by excessive inflammation within the CNS (1–3, 12, 13). It is now appreciated that resident glial cell expression and protein production of PRRs contributes to the recognition of and innate immune response to invading pathogens (10, 14, 15). Our data builds upon previous reports that RIG-I can detect bacterial nucleic acids in peripheral immune and non-immune cells by demonstrating that bacterial challenge induces RIG-I protein production in glia, suggesting a broader role in surveillance of the CNS (41, 45–47, 60–62). Our data show that RIG-I protein is constitutively produced in both human microglia and murine microglia and astrocytes and that such protein production is further upregulated following challenge with *Neisseria meningitidis* but not *Streptococcus pneumoniae*. The observed differences in RIG-I protein production following *N. meningitidis* and *S. pneumoniae* challenge suggest that RIG-I-mediated responses in glia are pathogen-specific. This pathogen specificity may reflect fundamental differences in how these bacteria interact with and are recognized by glial cells. While both the Gram-negative bacteria, *N. meningitidis* and Gram-positive bacteria, *S. pneumoniae* can adhere to and be internalized by host cells, these pathogens express distinct adhesins that can impact internalization. Additionally, each of these pathogens can release membrane vesicles composed of pathogen-specific lipids, sugars, proteins, and nucleic acids that serve as pathogen associated molecular patterns recognized by host PRRs (24, 63–66). These distinct host-pathogen interactions likely influence the extent to which bacterial nucleic acids become available for RIG-I-mediated recognition, shaping downstream immune responses.

Importantly, we demonstrate that RIG-I mediated detection of *N. meningitidis* and *S. pneumoniae* stimulates glial cell production of inflammatory and type I IFN immune mediators. Notably, human and murine glial cell types produced IFN-β in response to *N. meningitidis* and, to a lesser extent, *S. pneumoniae*, suggesting differential activation to relevant CNS pathogens (15, 20). While prior studies have shown glial IFN responses to isolated bacterial nucleic acids, our data demonstrate that in the context of infection, bacterial pathogens can elicit IFN-β production in a RIG I-dependent manner (48) as demonstrated by the sensitivity of these responses to siRNA knockdown of RIG-I.

Type I IFNs, including IFN-β, are key mediators of innate immunity that have been shown to exert broad antiviral effects by inducing the mRNA expression and protein production of ISGs (56, 57, 67). However, in the context of bacterial infection, type I IFNs have been shown to play complex and dichotomous roles (67–70). In some settings, they contribute to host protection by restricting intracellular bacterial replication and enhancing immune cell recruitment (71–73). On the other hand, they have also been shown to contribute to the exacerbation of disease by suppressing proinflammatory responses or impairing bacterial clearance (74–76). Our data support a protective role for RIG-I-stimulated IFN-β production by resident brain cells, as siRNA-mediated knockdown of RIG-I significantly reduces IFN-β production and consequently leads to a significant increase of *N. meningitidis* and *S. pneumoniae* burden in glial cells.

Additionally, we show that RIG-I activation leads to the induction of the ISGs, IFIT-1 and IFIT-3. These ISGs are widely known for their antiviral effects (77, 78); however, emerging studies indicate that several ISGs, including viperin, IFITMs, and others, can restrict intracellular bacterial replication (79–81). Consistent with these findings, we observed that IFIT-1 and IFIT-3 were upregulated in glial cells in response to both *N. meningitidis* and *S. pneumoniae* and that the observed induction was dependent on RIG-I activation. Our experiments employing siRNA knockdown of RIG-I or pharmacological inhibition of IFNAR signaling demonstrate that RIG-I stimulates IFN-β production by glial cells, which in turn promotes the induction of ISGs via IFNAR signaling. Furthermore, we show that IFNAR-mediated induction of ISGs is necessary to limit bacterial viability as pharmacological inhibition of IFNAR signaling increases bacterial burden, supporting that type I IFN production and signaling are protective during bacterial CNS infection.

Collectively, our results demonstrate a protective role for RIG-I-stimulated type I IFNs in contributing to the restriction of both *N. meningitidis* and *S. pneumoniae* in glial cells. Importantly, this suggests that modulation of IFN signaling pathways in the CNS may offer therapeutic benefit. Excitingly, we demonstrate that targeting RIG-I using established synthetic agonists can be employed to enhance protective IFN responses in glia, leading to further restriction of bacterial burden during *N. meningitidis* and *S. pneumoniae* infection. Our data support that priming glial cell types with established nucleic acid ligands enhances antimicrobial responses to relevant bacterial pathogens. These findings highlight the therapeutic potential of leveraging innate immune signaling pathways to enhance host defenses in the CNS. While this study focused on canonical RIG-I agonists, our previous work has shown that structured nucleic acid nanoparticles (NANPs) can also activate RIG-I and elicit IFN production in glial cells (48). These findings demonstrate that both conventional and novel agonists have the potential to augment protective glial responses during bacterial CNS infections. Collectively, these results establish RIG-I as a key innate immune sensor for relevant CNS pathogens with implications for immunomodulation during bacterial meningitis. Further investigation into RIG-I-driven signaling and the development of targeted RIG-I agonists may inform new strategies for controlling CNS infections.

## Data Availability

The original contributions presented in the study are included in the article/**Supplementary Material**. Further inquiries can be directed to the corresponding author.

## Glossary

ANOVA: Analysis of variance
ATCC: American type culture collection
BBB: Blood-brain barrier
B-DNA: B-form deoxyribonucleic acid
CFUs: Colony-forming units
CNS: Central nervous system
CSF-1: Colony-stimulating factor-1
DMEM: Dulbecco’s modified Eagle’s medium
dsRNA: Double-stranded ribonucleic acid
EDTA: Ethylenediaminetetraacetic acid
ELISAs: Enzyme-linked immunosorbent assays
FBS: Fetal bovine serum
IFIT-1: Interferon-induced protein with tetratricopeptide repeats 1
IFIT-3: Interferon-induced protein with tetratricopeptide repeats 3
IFNAR: Interferon-alpha/beta receptor
IFN-β: Interferon-beta
IFNs: Type I interferons
ISGs: Interferon-stimulated genes
LPS: Lipopolysaccharide
L2K: Lipofectamine 2000
MOI: Multiplicity of infection
NLRs: NOD-like receptors
N. meningitidis: Nm, Neisseria meningitidis
NOD: Nucleotide-binding oligomerization domain
PAMPs: Pathogen-associated molecular patterns
polyI:C: Polyinosinic-polycytidylic acid
PRRs: Pattern recognition receptors
RIG-I: Retinoic acid-inducible gene I
RLRs: Retinoic acid-inducible gene I-like receptors
RPMI: Roswell park memorial institute medium
SEM: Standard error of the mean
siRNA: Small interfering RNA
S. pneumoniae: Sp, Streptococcus pneumoniae
TBK1: TANK-binding kinase 1
TLRs: Toll-like receptors
