# Supplementary material for: RIG-I drives protective type I interferon production by glial cells in response to Neisseria meningitidis and Streptococcus pneumoniae challenge
Source: Front Immunol. 2025 Nov 18;16:1692421. doi: 10.3389/fimmu.2025.1692421 (PMC12669104; doi:10.3389/fimmu.2025.1692421)
Supplement: Supplementary file 1 [file DataSheet1.pdf]

Supplementary Material

1 Supplementary Data

1.1 Supplementary Figure 1

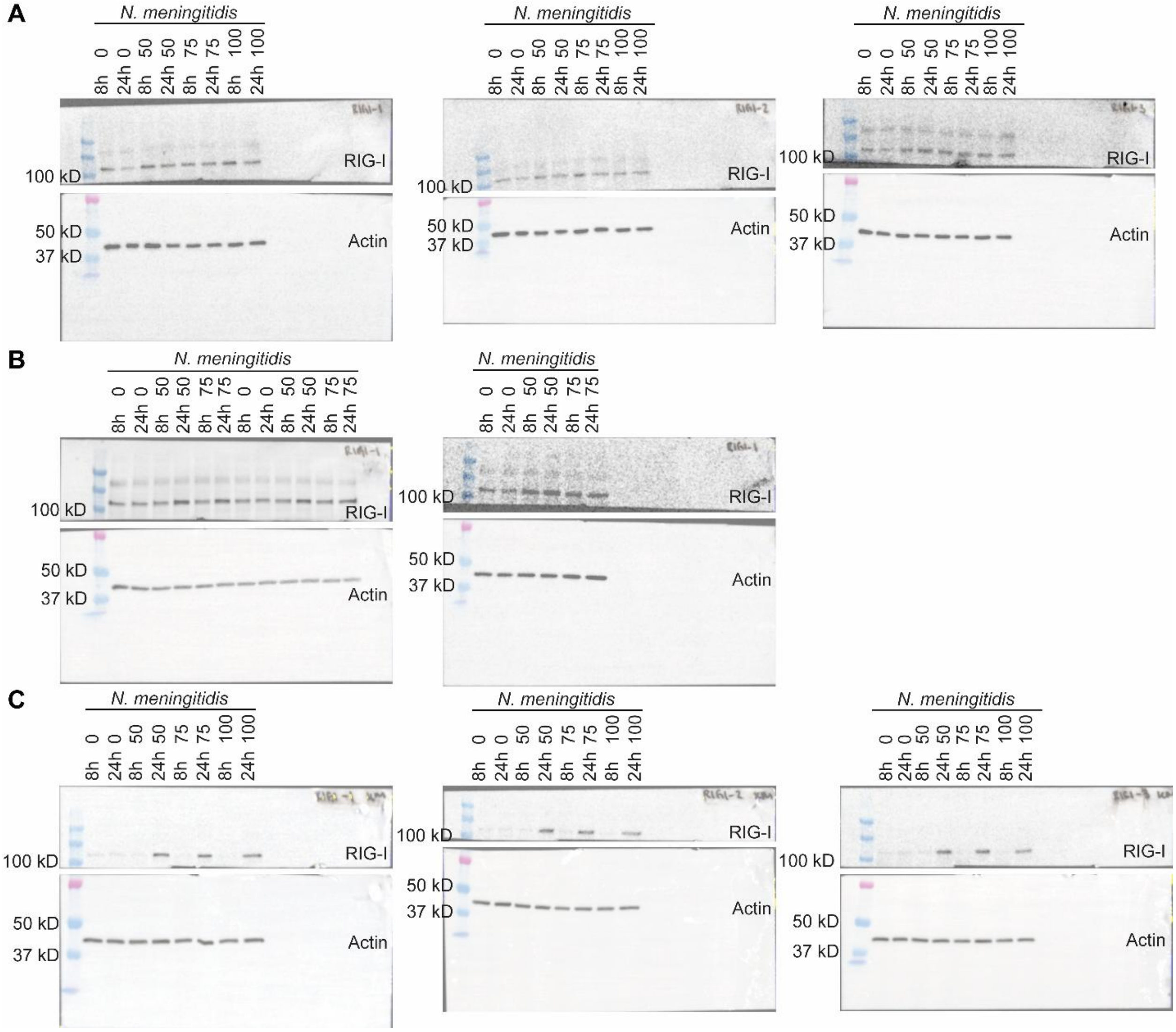

Supplemental Figure 1: Primary murine astrocytes (A), primary murine microglia (B), and human microglia (hHμC20) (C) were uninfected (0) or infected with *N. meningitidis* at MOIs of 50:1, 75:1, or 100:1. At 8 and 24 hours post-infection, expression of RIG-I (102 kDa) was assessed by immunoblot analysis and normalized to β-actin levels.

1.2 Supplementary Figure 2

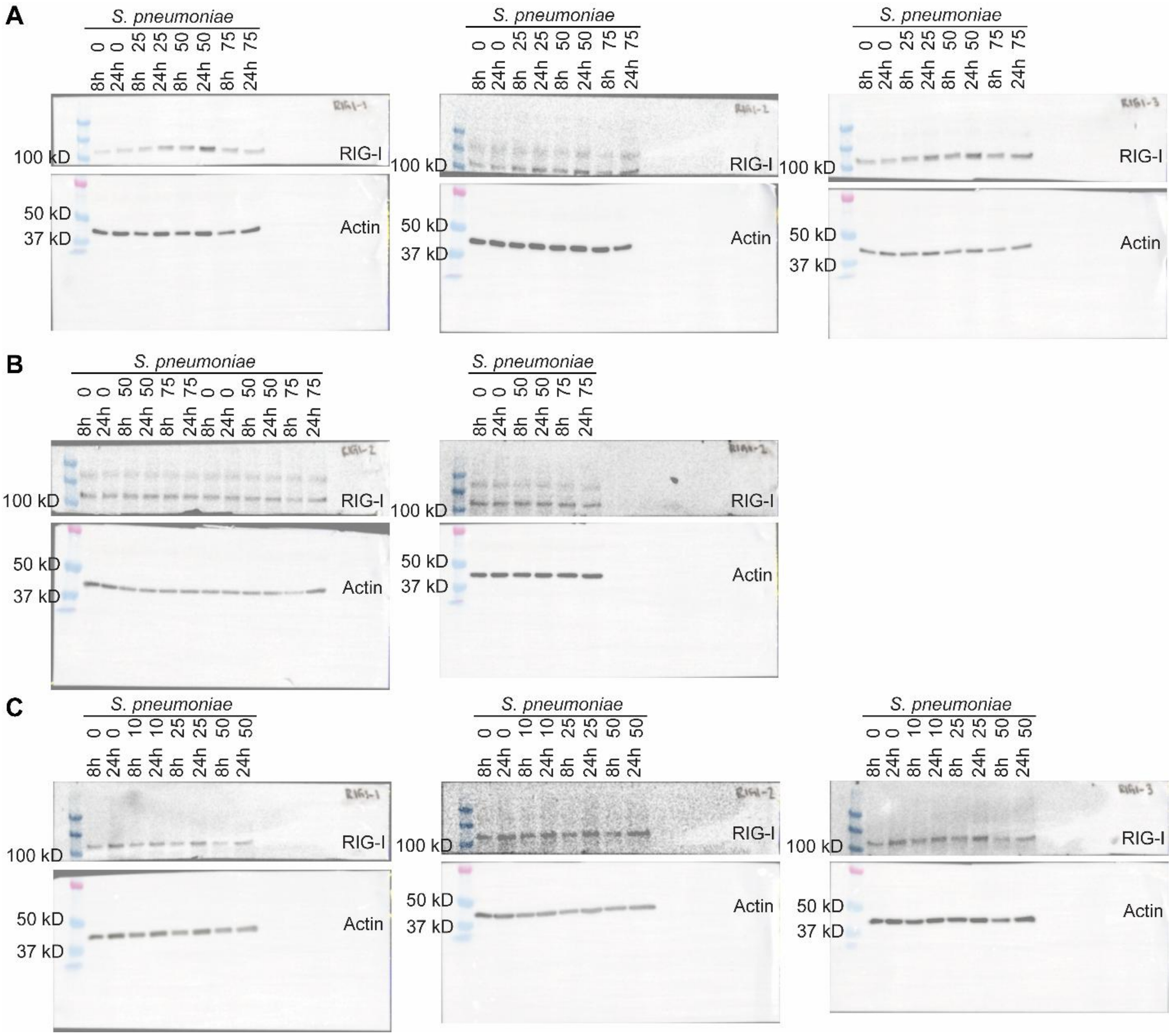

Supplemental Figure 2: Primary murine astrocytes (A), primary murine microglia (B), and human microglia (hHμC20) (C) were uninfected (0) or infected with *S. pneumoniae* at MOIs of 10:1, 25:1, 50:1 or 75:1. At 8 and 24 hours post-infection, expression of RIG-I (102 kDa) was assessed by immunoblot analysis and normalized to β-actin levels.

1.3 Supplementary Figure 3

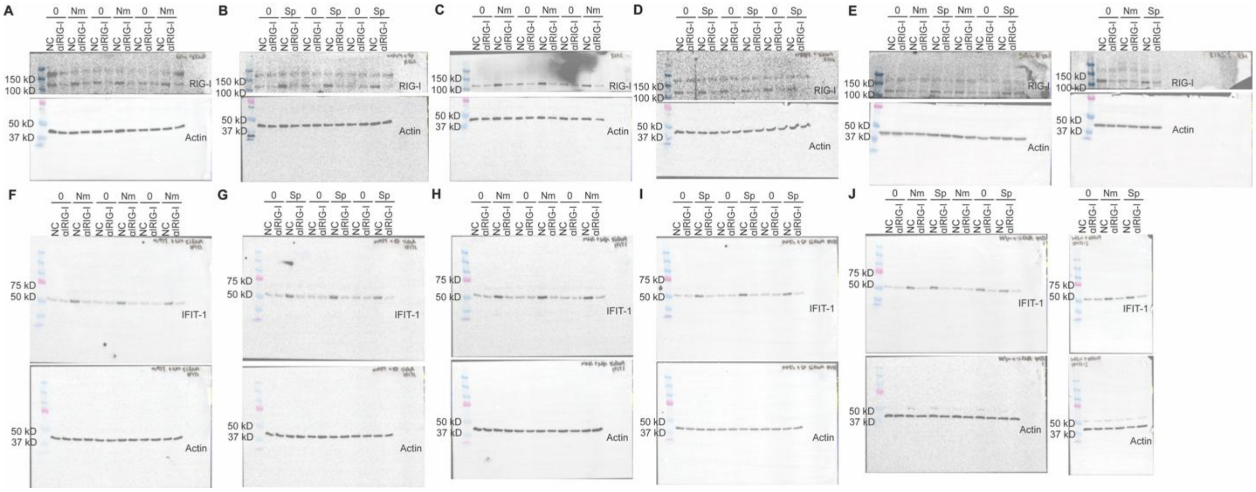

Supplemental Figure 3: Primary murine astrocytes (A-B, F-G), primary murine microglia (C-D, H-I) and human microglia (hHµC20) (E and J) were transfected with siRNA (5nM or 10nM) directed against RIG-I exposed to control siRNA (NC) using RNAiMAX. Cells were then left uninfected (0) or challenged with *N. meningitidis* αor *S. pneumoniae* (MOI of 50:1). At 24 h post-infection, expression of RIG-I (102 kDa) was assessed by immunoblot analysis in primary murine astrocytes (A-B), primary murine microglia (C-D), and human microglia (E) and normalized to β-actin levels. Additionally, expression of IFIT-1 (56 kDa) was assessed by immunoblot analysis in primary murine astrocytes (F-G), primary murine microglia (H-I), and human microglia (J) and normalized to β-actin levels.

1.4 Supplementary Figure 4

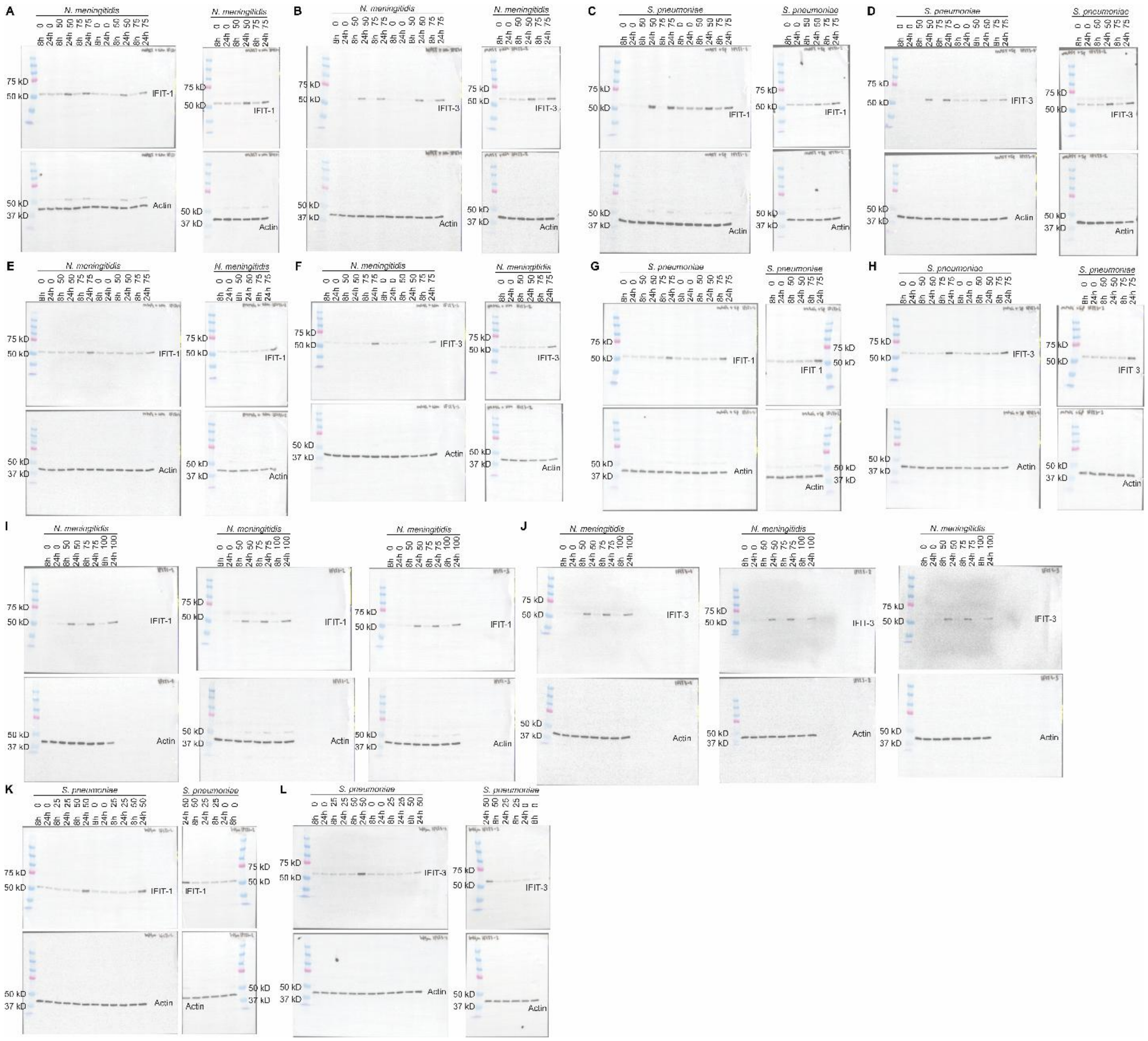

Supplemental Figure 4: Primary murine astrocytes (A-D) primary murine microglia (E-H) and human microglia (hHμC20) (I-L) were then left uninfected (0) or challenged with *N. meningitidis* or *S. pneumoniae* (MOI of 50:1). At 24 h post-infection, expression of IFIT-1 (56 kDa) and IFIT-3 (60-65 kDa) was assessed by immunoblot analysis in primary murine astrocytes (A-D), primary murine microglia (E-H), and human microglia (I-L) and normalized to β-actin levels.

1.5 Supplementary Figure 5

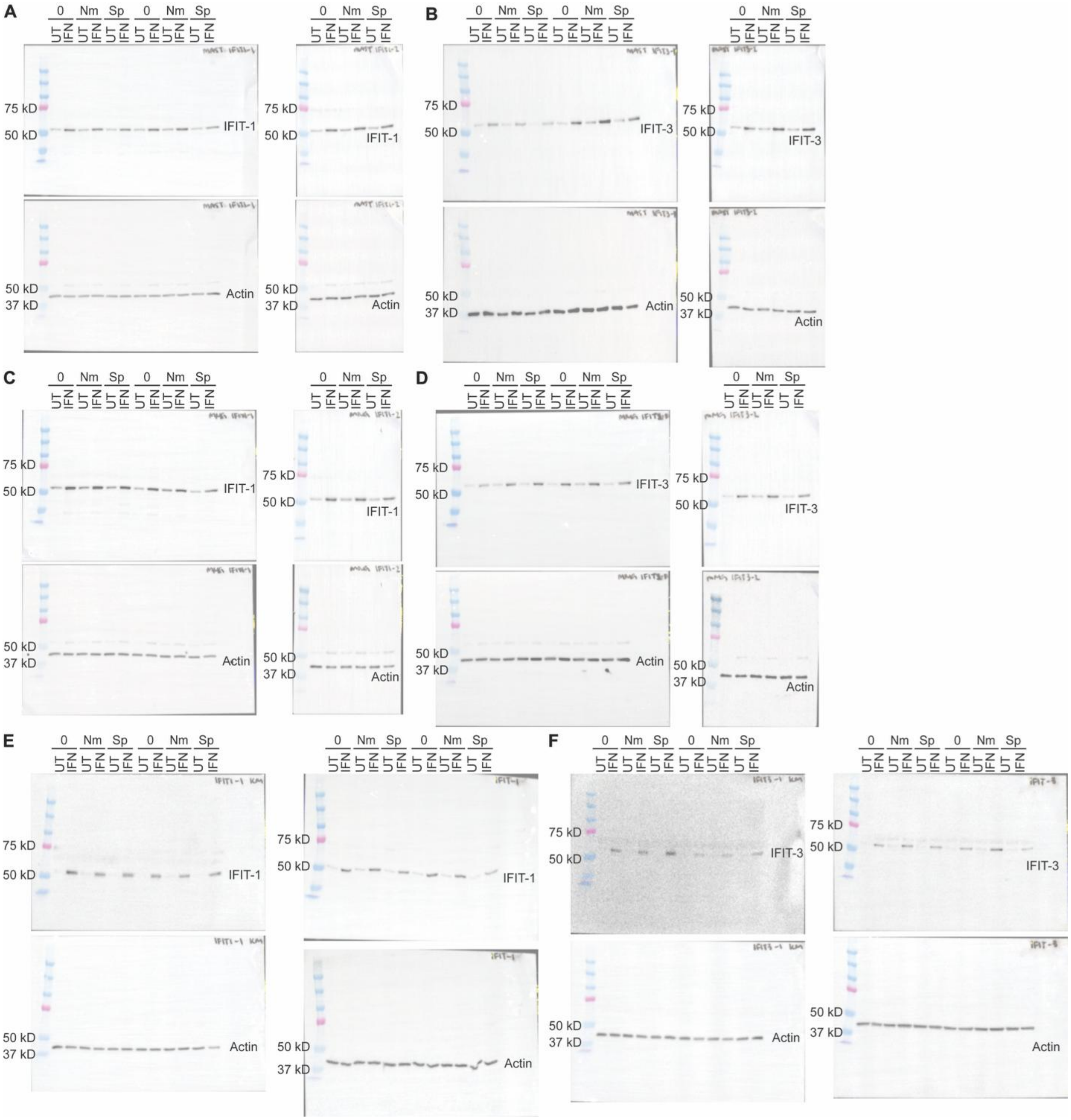

Supplemental Figure 5: Primary murine astrocytes (A-B) primary murine microglia (C-D) and human microglia (hHµC20) (E-F) were left untreated or treated with exogenous IFN- β. Cells were then left uninfected (0) or challenged with *N. meningitidis* or *S. pneumoniae* (MOI of 50:1). At 6 h post-infection, expression of IFIT-1 (56 kDa) and IFIT-3 (60-65 kDa) was assessed by immunoblot analysis in primary murine astrocytes (A-B), primary murine microglia (C-D), and human microglia (E-F) and normalized to β-actin levels.

1.6 Supplementary Figure 6

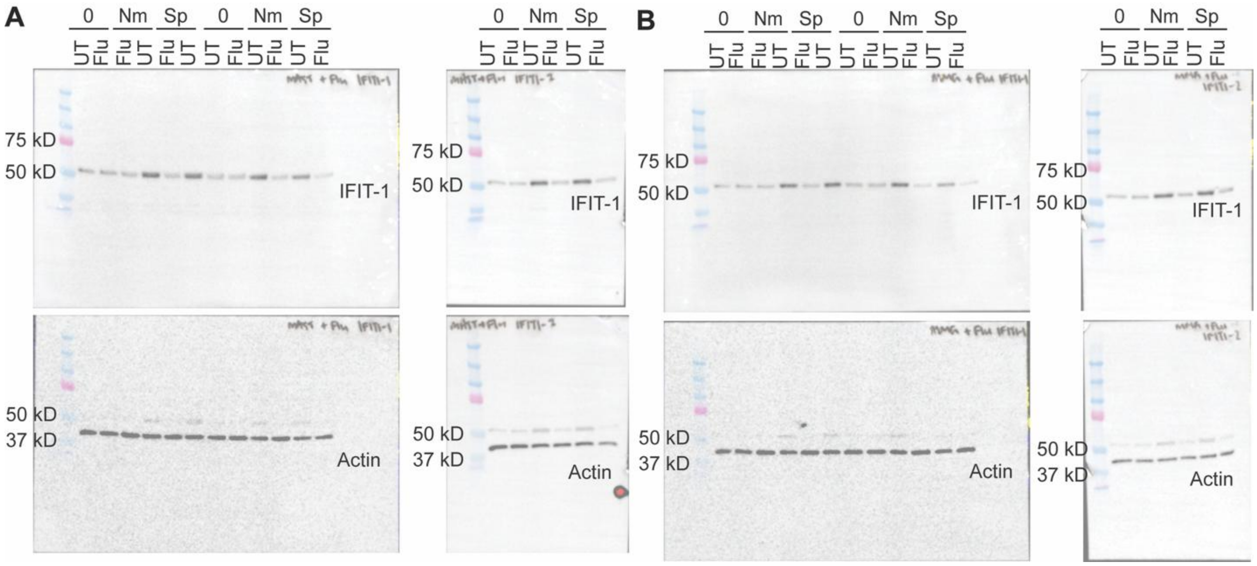

Supplemental Figure 6: Primary murine astrocytes (A) and primary murine microglia (B) left untreated or treated with Fludarabine. Cells were then left uninfected (0) or challenged with *N. meningitidis* or *S. pneumoniae* (MOI of 50:1). At 24 h post-infection, expression of IFIT-1 (56 kDa) was assessed by immunoblot analysis in primary murine astrocytes (A) and primary murine microglia (B) and normalized to β-actin levels.

1.7 Supplementary Figure 7

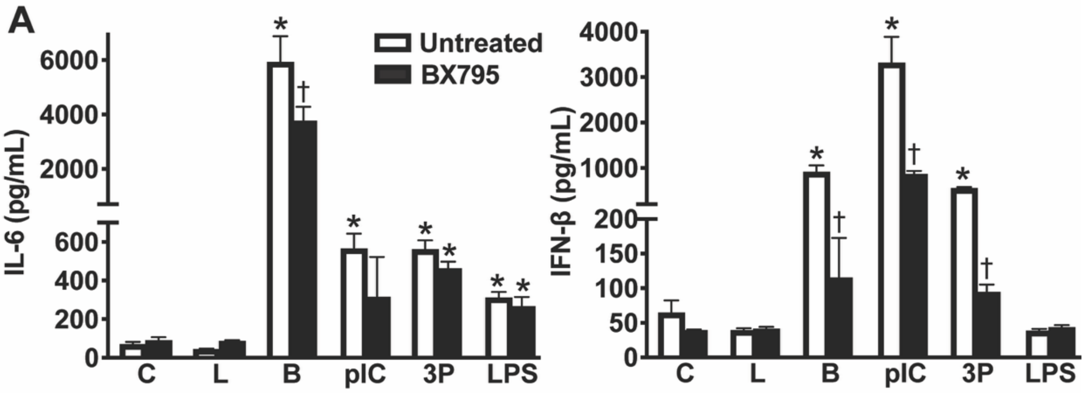

Supplemental Figure 7: Human microglia (hHµC20) were left untreated or treated with BX795. Human microglia (hHµC20) were left untreated or treated with a TBK1/IKKε inhibitor (BX795) for 2 h prior to stimulation with ligands including B-DNA (B), polyinosinic-polycytidylic acid (pIC), and 5'-triphosphosphate double-stranded RNA (3P) using Lipofectamine 2000 (L), or exogenously treated with lipopolysaccharide (LPS) for 2 h. Cells were next left uninfected. At 8 h post-infection, IL-6 and IFN-β production was assessed by specific capture ELISAs.
